# Supplementary material for: Complexome profiling of the Chlamydomonas psb28 mutant reveals TEF5 as an early PSII assembly factor
Source: Plant Cell. 2025 Mar 17;37(6):koaf055. doi: 10.1093/plcell/koaf055 (PMC12164590; doi:10.1093/plcell/koaf055)
Supplement: koaf055_Supplementary_Data [file koaf055_supplementary_data.zip › psb28_interactive.zip!/plots/intensity/qualityPlots/peptides/_Cre14.g626750.t1.2.html]

### Cre14.g626750.t1.2

Trivial name:   
SeqCoverage [median]: 33.90  
Mass: 20.42 kDa  
  
 

| Sequence | MissedCleavage | Mass [Da] | wtIntensity | muIntensity | Score |
| --- | --- | --- | --- | --- | --- |
| DTYSVPEGVFTAANEK | 0 | 1726.81 | 0.00 | 7499.02 | 72.29 |
| EMPETINGR | 0 | 1045.49 | 0.00 | 3155.78 | 77.92 |
| FLESSDVKLEELPAYK | 1 | 1866.96 | 13375.78 | 114397.26 | 157.23 |
| GTQGDYLSALK | 0 | 1151.58 | 312.70 | 7420.59 | 110.84 |
| LEELPAYK | 0 | 961.51 | 0.00 | 305.21 | 84.24 |
| TYLQPYVESELFK | 0 | 1615.81 | 4622.59 | 4969.33 | 116.55 |
|  |  |  | 18311.1 | 137747.2 |  |
